# Molecular basis of anti-CD36 alloimmunization: transcriptomic evidence linking intronic splice-site mutations to CD36 loss-of-function

**DOI:** 10.3389/fimmu.2026.1945011

**Published:** 2026-09-28

**Authors:** Hui Ren, Hong Luo, Haoqiang Ding, Xin Ye, Mingqin Mai, Xinnian Li, Dawei Chen, Yalin Luo, Shengxue Luo, Xiuzhang Xu, Yongmei Nie, Jing Wu, Huaqin Liang, Wenjie Xia

**Affiliations:** 1 Institute of Blood Transfusion, Guangzhou Blood Center, Guangzhou, China; 2 The Key Medical Laboratory of Guangzhou, Guangzhou, China; 3 Medical Genetics Center, Guangdong Women and Children Hospital, Southern University of Science and Technology, Guangzhou, China; 4 Guangzhou Forevergen Medical Laboratory, Guangzhou, China

**Keywords:** aberrant transcripts, CD36, intron mutation, splice-site variant, type I deficiency

## Abstract

**Background:**

CD36 deficiency is a genetic disorder that can lead to fetal/neonatal alloimmune thrombocytopenia or platelet transfusion refractoriness. While CD36 deficiency caused by exonic mutations are well established, the RNA splicing mechanism by intronic mutations remains poorly defined.

**Methods:**

CD36 deficiency patients were analyzed by long-read sequencing and haplotype analysis. RNA sequencing and allele-specific analysis validated mutation–splicing associations in patients. CD36 aberrant transcript plasmids were constructed and assessed by flow cytometry. Exome sequencing of healthy donors determined mutation frequencies.

**Results:**

We found that population screening revealed a carrier frequency of approximately 2/800 for c.430-1G>C mutation and ~3/800 for c.429 + 3*ins*G mutation among healthy Chinese donors. This study includes seven type I CD36 deficiency patients with PTR or FNAIT. Allele-specific analysis confirmed mutant allele enrichment in aberrant transcripts (*P* = 0.024). Long-read sequencing showed that one patient only had two intronic mutations (c.430-1G>C and c.429 + 3*ins*G). Indicating intronic mutation was significantly linked to type I CD36 deficiency. Specifically, c.430-1G>C mutation induced aberrant exon 6 splicing, resulting in 17-bp pseudo-intron insertion or complete exon 6 skipping (28.6% or 30.3% skipped); the c.429 + 3*ins*G mutation caused exon 5 skipping (38.3% skipped). Functional assays showed that the c.430-1G>C mutation led to complete loss of CD36 protein expression.

**Conclusion:**

This study establishes that intronic mutations alone can also induce CD36 deficiency by impairing mRNA splicing, thereby extending the known mutational landscape of CD36 deficiency and reinforcing the necessity of screening intronic mutations in diagnostic and pathogenetic investigations.

## Introduction

CD36 (also known as GPIV) is expressed on platelets, monocytes, and endothelial cells (1). Type I deficiency is characterized by reduced CD36 levels in platelets and monocytes, whereas type II deficiency is characterized by a lack of CD36 surface expression on platelets (2). Individuals with type I CD36 deficiency may develop iso-antibodies through blood transfusion, pregnancy, and transplantation, which are associated with platelet transfusion refractoriness (PTR), fetal/neonatal iso-immune thrombocytopenia (FNAIT), and transfuse-related acute lung injury (TRALI) (3–7). The frequency of these pathologies varies significantly across regions, countries, and ethnic groups, with extremely low incidence of CD36 deficiency in American populations (<0.4%) (8) and higher incidence in African (<7%) and relatively high in Asians (3%–10%) (9–13). In China, 0.2%–0.5% of the population has type I deficiency and 1.2%–2.0% has type II deficiency (14, 15).

The CD36 gene (7q11.2) comprises 15 exons, with coding sequence spanning exons 3–14 (16). More than 20 coding mutations cause type I deficiency (17), with c.329–330*del*AC, c.1228–1239*del*ATTGTGCCTATT, and c.268C>T accounting for approximately 90% of cases (18–21). The mutations of type II CD36 deficiency remains poorly characterized (9); nevertheless, several variants have been reported, such as c.478C>T (11, 14), c.1344*ins*TCTT, and c.1163A>T (9). Furthermore, several intronic mutations have also been associated with CD36 deficiency—for example, 1200-13A>G in intron 12 (22) and (TG) 12 TAA (TG) 2 repeat in intron 3 (23) are each associated with CD36 type I deficiency, whereas c.120 + 1G>T in intron 3 is associated with CD36 type II deficiency (18, 24).

Third-generation sequencing (TGS), which can span entire genes and complex genomic regions, has demonstrated significant utility in diagnosing rare genetic diseases and detecting previously hidden aberrations missed by NGS (25). A recent work from our group has highlighted TGS as a key tool for elucidating the genetic landscape of CD36 deficiency in which single-molecule long-read sequencing of genomic DNA was used to explore its genetic mechanisms (20). Although over 30,000 variants of the CD36 gene are listed in the dbSNP database, not all of these mutations lead to altered CD36 expression (26).

Nowadays, the majority of mutation analyses have concentrated on exonic regions, whereas intronic mutations have rarely been reported. Restrictions of exome sequencing, inaccurate splicing prediction, and scarce patient tissue samples render most intronic variants clinically unclassified. Recent studies have shown that intronic mutations can activate splice sites or disrupt splicing silencers, leading to pseudo exon inclusion or exon skipping (27). We performed patient-based allele-specific splicing assays to overcome computational and minigene limitations, delineating splicing defects of intronic variants and their clinical relevance. Based on these findings, we explored whether intronic mutations contribute to the pathogenesis of CD36 deficiency by performing haplotype sequencing in Chinese patients with CD36 deficiency-induced PTR or FNAIT. Notably, one patient only carried intronic mutation in intron 5, which resulted in skipping of exon 5 or exon 6. Collectively, our findings expand the current understanding of CD36 deficiency and underscore the necessity of incorporating intronic regions into routine clinical diagnostics.

## Materials and methods

### Ethics statement

This study was performed according to the principles of the Declaration of Helsinki and was approved by the Guangzhou Blood Center Ethics Committee (no. 22N00789). All participants provided written informed consent.

### Study subjects, grouping, and haplotype analysis

Between 2015 and 2022, the Guangzhou Blood Center recruited seven unrelated Chinese patients for whom type I CD36 deficiency had been diagnosed according to the absence of CD36 expression on both platelets and monocytes and the presence of anti-CD36 alloantibodies; three patients presented with FNAIT and four with PTR (**Table 1**). All were negative for erythrocyte antibodies as assessed by the Coombs test. Long-read sequencing was used to phase CD36 mutations and determine their allelic configuration as described (18). In addition, healthy blood donors (594 male and 206 female; mean age 35.5 ± 9.6 years) were randomly recruited from the Guangzhou Blood Center (**Table 2**). Approximately 97% of these donors were self-reported Han Chinese.

**Table 1:** Genotypic details of seven patients.

Patients	Age/sex	Type	Antibodies	Clinical symptoms	Disease	CD36 gene mutation				Related publication
						Intronic variants		Exonic variants		
1	30/F	I	Anti-CD36 antibodies, anti-HLA I antibodies	CCI < 7,000, PPR < 20%	PTR			c. 380 C>T		[18]
									c. 1156 C>T	
2	35/F	I	Anti-CD36 antibodies	CCI < 7,000, PPR < 20%	PTR			c.1163 A>T		[18]
									c. 1156 C>T	
3	48/F	I	Anti-CD36 antibodies, anti-HLA I antibodies	CCI < 7,000, PPR < 20%	PTR		c.430-1G>C			Current report
						c.429 + 3 *ins* G				
4	46/F	I	Anti-CD36 antibodies	CCI < 7,000, PPR < 20%	PTR			c. 380 C>T		[18]
									c. 329–300 *del* AC	
5	33/F	I	Anti-CD36 antibodies, anti-HLA I antibodies	G2P0A6 Hydrops fetalis, anemia	FNAIT			c. 329–300 *del* AC		[18]
									c. 329–300 *del* AC	
6	32/F	I	Anti-CD36 antibodies, anti-HLA I antibodies	G7P2A5 Hydrops fetalis, anemia	FNAIT			c. 329–300 *del* AC		[18]
									c. 329–300 *del* AC	
7	30/F	I	Anti-CD36 antibodies	G2P1A1 Hydrops fetalis, anemia	FNAIT		c.430-1G>C			Current report
									c. 329–300 *del* AC	

CCI, correct count increment; FNAIT, fetal/neonatal alloimmune thrombocytopenia; PPR, platelet recovery rate; PTR, platelet transfusion refractoriness.

**Table 2:** Detailed information of 800 blood donors.

	Healthy donor	
	Female (*n* = 206)	Male (*n* = 594)
Age in years (mean ± SD)	35.49 ± 9.59	35.45 ± 9.61
Blood types		
A	64	181
B	62	143
AB	12	39
O	68	231
Ethnic group		
Han	203	576
Ethnic minority	3	18

### Transcriptome sequencing and transcript scanning

Two patients from the discovery cohort who carried ≤1 previously reported exonic variant were selected for platelet/monocyte RNA-seq. CD36-positive donors, matched for age and sex, were used to establish baseline splicing patterns.

For mRNA sequencing, raw reads were quality-trimmed to remove adapters and low-quality bases (Phred score < 20) using Trimmomatic v0.39 (28). The cleaned reads were aligned to the human reference genome (GRCh38) using STAR v2.7.9a with parameters optimized for detecting splice junctions (e.g., “--outFilterMismatchNoverLmax 0.1”, “--alignIntronMin 20”, “--alignIntronMax 1000000”) (29). The aligned reads were output in BAM format and sorted using SAMtools v1.16 (30). Duplicate reads resulting from PCR amplification were marked using Picard Tools v2.27.0 (“MarkDuplicates”).

To reveal the influence of genetic variants of CD36, the sequencing reads spanning the region containing the genetic variants were verified, and the splice junctions were detected using a custom script in Perl. For the case of Patient 3, samtools view and the IGV browser were used to enumerate the reads within the genomic coordinates of c.329-330*del*AC and c.430-1G>C (GRCh38, chr7:80,661,110-80,662,989) in the RNA-BAM file. The reads were then split into two groups: (i) “normally spliced”, those that span the exon 5–exon 6 junction (i.e., no retention, no skipping) and (ii) “aberrantly spliced”, those that either (a) include the 17-bp pseudo-intron between exon 5 and exon 6 or (b) skip exon 6 completely (junction reads exon 5→exon 7). A 2 × 2 contingency table was constructed with rows—DNA allele (ref G vs mutant C) and columns—splicing outcome (normal vs aberrant). The odds ratio (OR) and one-tailed *P*-value were calculated using Fisher’s exact test; OR >1 and *P* < 0.05 were considered significant.

The same pipeline of RNA-seq and analyses for other CD36-positive donors was conducted as background controls. Allele-specific validation was extended to patient 3 who carries c.430-1G>C on one allele and c.429 + 3*ins*G on the other. The RNA-seq reads were phased with long-range gDNA haplotypes as described above. To quantify the relative contribution of each allele to global mis-splicing, the percent-spliced-in (PSI) and percent-skipped (PSK) values were calculated for every exon–exon link:

PSI = inclusion_reads/(inclusion_reads + skipping_reads)

PSK = 1 − PSI

Background distributions of PSI/PSK were derived from 10 CD36-positive donors (Norm01–Norm10); a *Z*-score >2 or FDR <0.1 was considered significant. These thresholds were adopted based on established practices in large-scale RNA splicing analyses to balance sensitivity and specificity while minimizing false-positive discoveries (27).

### Construction of overexpression and mutation plasmids

Flag-tagged versions of the wild type or mutant CD36 mRNA transcript coding sequence were synthesized by a biological product company (Cookgene Bio Ltd., China). The HEK-293T (293T) cells were from Guangzhou Xinyuan Technology Co., Ltd. (Guangzhou, China, cat. no. XY-0418).

For the assessment of CD36 protein expression, 293T cells were seeded in six-well plates at a density of 5 × 10^5^ cells per well and cultured in Dulbecco’s Modified Eagle Medium (DMEM) supplemented with 10% fetal bovine serum (FBS) and 1% penicillin–streptomycin at 37 °C containing 5% CO_2_. When the cells reached 70%–80% confluence, they were transfected with the verified Flag-tagged wild-type (Normal CD36) or splicing-variant (c.430-1G>C_17insert or c.430-1G>C_Δexon6) plasmids using Lipofectamine 2000 (Thermo Fisher Scientific, USA). After 48 h of transfection, the cells were harvested by trypsinization and washed twice with phosphate-buffered saline (PBS). The cells were incubated with FITC-labelled anti-CD36 for 30 min at room temperature in the dark. After three additional washes with PBS, the expression level of Flag-tagged CD36 protein was detected and quantified by flow cytometry (BD FACS Canto II, BD Biosciences, USA).

## Results

### One patient with type I CD36 deficiency exclusively caused by intronic mutations

To identify the etiology of type I CD36 deficiency, we analyzed the genotypic and clinical features of seven female patients aged 30 to 48 years with CD36-related alloimmune disorders who were all confirmed to have type I CD36 deficiency. Among them, four presented with platelet transfusion refractoriness (PTR) and three with fetal/neonatal alloimmune thrombocytopenia (FNAIT) (**Table 1**). For PTR patients, the consistent clinical manifestations were corrected count increment (CCI) <7,000 and platelet recovery rate (PPR) <20%. Among the PTR cases, two patients presented with both anti-CD36 and anti-HLA class I alloantibodies, whereas another two patients had isolated anti-CD36 antibodies without detectable anti-HLA class I antibodies. In contrast, FNAIT patients clinically manifested with hydrops fetalis and anemia. Specifically, two FNAIT patients with combined anti-CD36/anti-HLA I antibodies presented obstetric histories of Gravida 2, Para 0, Abortus 6 (G2P0A6) and G7P2A5, respectively. The remaining FNAIT patient with isolated anti-CD36 antibodies presented obstetric histories of G2P1A1.

Analysis of *CD36* gene sequences (exons 1–15) from each patient (**Table 1**) identified several pathogenic mutations, including exonic and intronic mutations. Among the seven patients, five carried dual exonic mutations, which included recurrent mutations such as c.380C>T, c.1156C>T, c.1163A>T (predominantly detected in PTR patients), and c.329-330*del*AC (predominantly detected in both PTR and FNAIT patients). The remaining two patients exhibited divergent mutational landscapes, with patient 3 representing a unique case bearing only intronic variants without any exonic mutations. Patient 3 carried two intronic variants located within intron 5: c.430–1G>C and c.429 + 3*ins*G (**Supplementary Figure S1**). Patient 7 possessed a single exonic mutation (c.329–330*del*AC) alongside the intronic variant c.430–1G>C in intron 5. Notably, CD36-specific antibodies were detectable in the two patients who concurrently exhibited complete loss of CD36 expression on both platelets and monocytes (**Supplementary Figure S2**). The intronic variants c.430–1G>C and c.429 + 3*ins*G identified in patient 3 are described here for the first time; all other variants have been documented in prior literature. This finding suggests that these intronic alterations may serve as critical drivers underlying CD36 deficiency.

### RNA sequencing analysis of CD36 intronic mutation and allele-specific validation of aberrant splicing

To elucidate the splicing regulatory mechanism driven by intronic mutations, we conducted a focused analysis in CD36 deficiency patients with confirmed intronic mutations (**Table 1**). For patient 3, carrying c.430-1G>C and c.429 + 3*ins*G intronic mutations displayed a high incidence of aberrant CD36 splicing (**Figure 1**). Among 13 RNA-seq reads that simultaneously covered the c.430-1G>C position and the exon 5–exon 6 junction region, six supported aberrant splicing (17-bp pseudo-intron retention or exon 6 skipping) and seven supported normal splicing (**Figure 1A**). The seven normal reads all carried the reference G allele, while four of the aberrant reads carried the mutant C allele (**Table 3**). Statistical analysis verified that the mutant C allele was significantly enriched among aberrant junction reads (odds ratio [OR] = 27.0, 95% confidence interval [CI]: 1.56 - ∞; *P* = 0.024, OR > 1), suggesting that aberrant splicing is allele specific. The infinite upper bound of the CI is a consequence of the zero count of mutant alleles in the normally spliced reads, reflecting a strong and exclusive association. This analytical approach is consistent with established methodologies for allele-specific splicing validation (27). The c.430-1G>C mutation, with specific focus on reads with 17-bp (TGACTTTGTTTTTGTAG) pseudo-intron retention before exon 6 or exon 6 skipping, ranged from ~0% to ~30%. Compared with normal CD36-positive donors, the ratio of the reads with insertions ranged from ~10% to ~50%. Furthermore, four reads with exon 5 skipping were identified (**Figure 1B**), which might be associated with the c.429 + 3*ins*G mutation. Moreover, four reads with exon 5 skipping were identified (**Figure 1B**), which might be associated with the c.429 + 3*ins*G mutation and constituted 38.3% of the assessed transcripts (**Table 4**, **Supplementary Table S1**). The allele-specific pattern suggests that the c.430-1G>C and c.429 + 3*ins*G change, rather than other co-existing exon variants, is likely to drive the aberrant splicing of CD36 exon 6 and exon 5.

**Figure 1:**
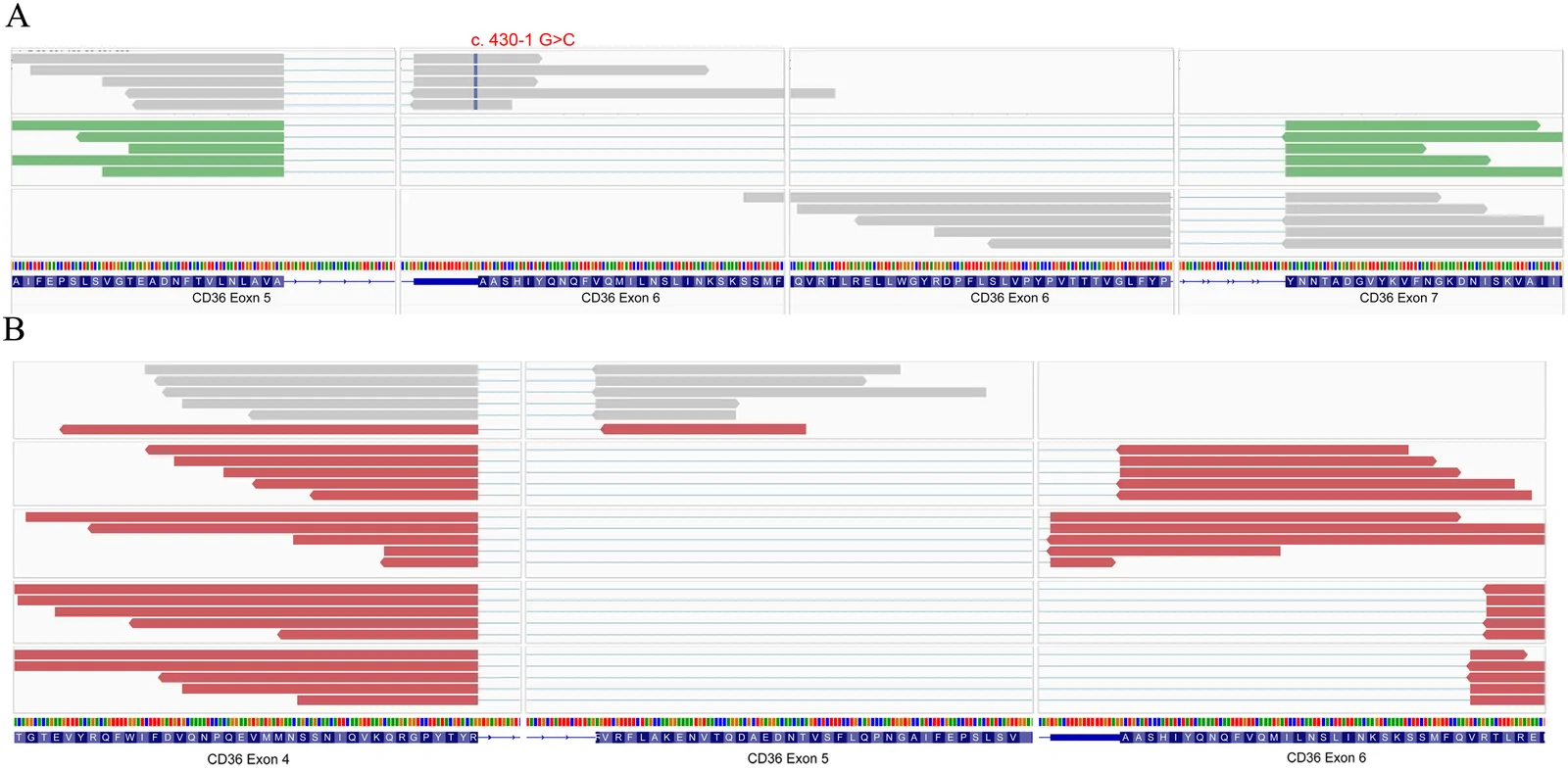
IGV browser for transcript reads of patient 3. **(A)** Transcript reads covered exons 5–7 from the platelets of patient 3. Reads with mutation c.430-1G>C is in gray (top five reads), and all of them are 17-bp (TGACTTTGTTTTTGTAC) pseudo-intron retention. Transcript reads skipping exon 6 were identified, which are presented in green. There were a few normal splicing transcripts read (gray) presented in bottom. **(B)** Moreover, for the platelets of patient 3, four types of transcript reads (presented in red) with exon 5 skipping were also identified. The normal transcript reads (gray) on top are also presented.

**Table 3:** RNA-seq reads results for G>C mutation in patient 3.

	Normal junction	Aberrant junction
Ref allele (with c.430-1 G)	7	2
Mut allele (with c.430-1 C)	0	4

**Table 4:** Percent-skipped values for every exon–exon link.

	Norm 1–10	Patient 3
Exon 5 skipping	0%–1%	38.3%
Exon 6 skipping	0%–0.5%	30.3%

Thus, intronic mutations c.430-1G>C and c.429 + 3*ins*G trigger allele-specific aberrant splicing of CD36, including pseudo-intron retention and exon 6 and exon 5 skipping, which contributed to the molecular pathogenesis of type I CD36 deficiency.

### Impaired CD36 expression caused by the c.430-1G>C intronic mutation

Flow cytometry analysis revealed distinct CD36 expression profiles among the three groups. In the wild-type CD36 group, a robust CD36 protein expression was detected on the cell surface, with a clear positive population observed (**Figure 2A**). In contrast, no specific CD36 expression was observed in cells carrying the c.430-1G>C_17insert variant (**Figure 2B**). The fluorescence histogram overlapped completely with that of the negative control, indicating a complete absence of CD36 protein. Similarly, cells expressing the c.430-1G>C_Δexon6 mutation also showed no detectable CD36 expression, with the signal remaining at background levels (**Figure 2C**). Neither splice-altering variant yielded any positive shift in fluorescence intensity.

**Figure 2:**
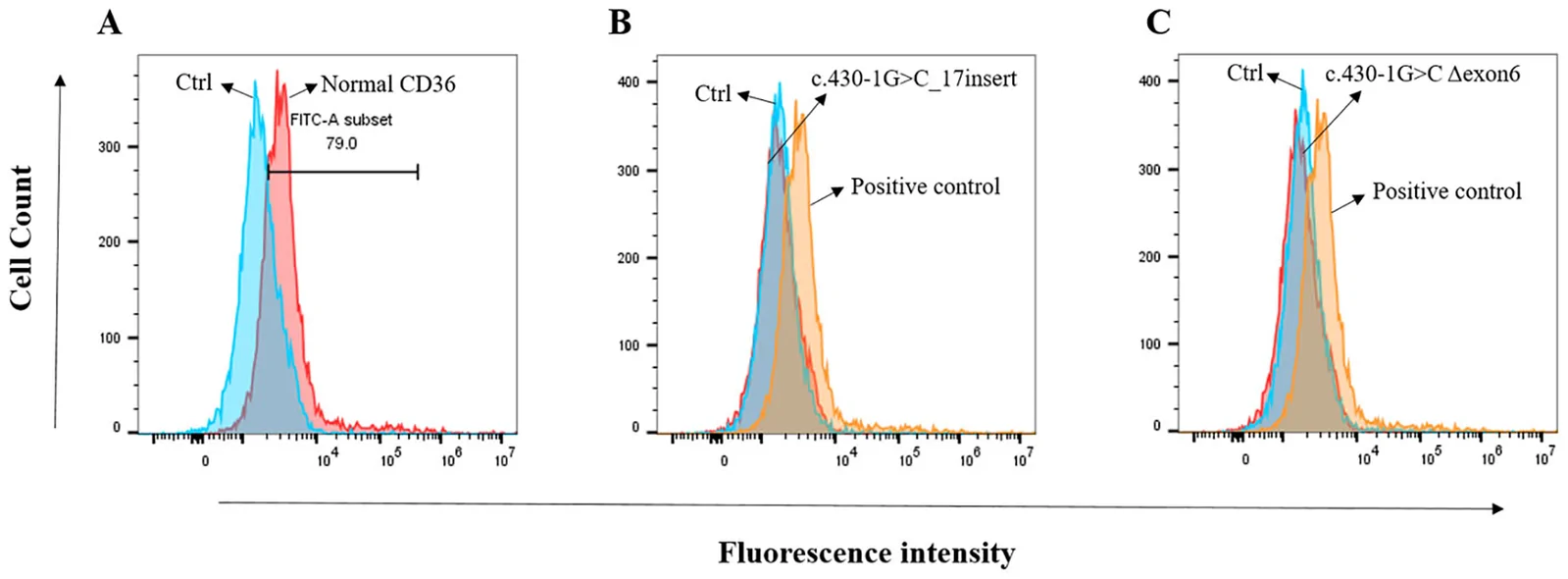
Analysis of CD36 expression after transfection of CD36 overexpression vectors. The CD36 mRNA transcript coding sequence was transferred into 293T cells. The expression of CD36 was detected by flow cytometry after transfection of the wild type (normal CD36; **(A)**), 17-bp insertion (c.430-1G>C_17insert; **(B)**), and exon 6 deletion (c.430-1G>C_Δexon6; **(C)**). The orange curve in **(B)** and **(C)** represents the positive control, which consists of 293T cells transfected with the wild-type CD36 expression vector, used to confirm antibody binding and assay functionality.

### Frequency of CD36 deficiency in Chinese blood donors

Exome sequencing of 800 Chinese blood donors identified two individuals (2/800) carrying a heterozygous mutation at the c.430-1G>C position and three individuals (3/800) carrying a heterozygous mutation at the c.429 + 3*ins*G position. Thus, the observed allele frequency of c.430-1G>C in this Chinese cohort was 0.25% (2/800) and that of c.429 + 3*ins*G was 0.375% (3/800). Notably, all identified mutations were heterozygous, indicating that each affected individual carried one normal allele and one mutated allele at the respective locus. For comparison, the reported carrier frequencies of these two mutations in global East Asian populations are 0.098% and 0.087%, respectively (gnomAD database). In the Chinese blood donor cohort, the carrier frequencies of CD36 c.430-1G>C and c.429 + 3*ins*G were higher than those in East Asian populations from gnomAD.

## Discussion

CD36 is a critical transmembrane glycoprotein that plays a central role in many key physiological processes (31–33). Clinical reports of PTR, FNAIT, and TRALI associated with CD36 antibodies continue to emerge. Although current evidence has linked the pathogenesis of CD36 deficiency mainly to homozygous or heterozygous mutations in exonic regions, the molecular mechanisms of CD36 deficiency resulting from intronic mutations remain poorly understood. To fill this gap, this study sought to preliminarily characterize the role of intronic mutations in CD36 deficiency, offering new insights into the genetic basis of the disease (34–36).

In Chinese patients with type I CD36 deficiency, we identified that one patient only had two novel intronic mutations in intron 5: c.430-1G>C and c.429 + 3*ins*G. Screening of an independent cohort of 800 healthy blood donors revealed that the frequencies of c.430-1G>C and c.429 + 3*ins*G were 0.25% and 0.375%, respectively, both higher than the carrier frequencies reported for the East Asian population from the Genome Aggregation Database (gnomAD) (0.098% and 0.087%, respectively). Although the frequencies of these two intronic mutations are relatively low in the Chinese population, they are approximately 2.5- or 4.3-fold higher than those observed in the broader East Asian population. These findings suggest that these two newly identified intronic mutations may play a potential role in the pathogenesis of type I CD36 deficiency, providing new research clues for further investigation into the genetic mechanisms underlying this disease.

The molecular basis of type I CD36 deficiency is as follows: c.329–330*del*AC causes a frameshift and premature stop codon (14, 37), c.1228–1239*del*ATTGTGCCTATT truncates the protein to a precursor by deleting four residues (14), and c.268C>T leads to Pro90Ser mutation, disrupting post-translational modification and leading to cytoplasmic degradation (15). Type II CD36 deficiency remains poorly characterized, and while some individuals lack detectable coding mutations (9), several variants have been functionally linked to this phenotype. The c.478C>T (Pro90Ser) mutation impairs post-translational maturation (11, 14), c.1344*ins*TCTT induces a frameshift and eliminates the stop codon, and c.1163A>T affects mRNA stability, reducing CD36 expression levels (9). Notably, another mutation located in the intronic region, c.429 + 4*ins*G, as one of the common pathogenic mutations causing type I CD36 deficiency, has been reported in the literature and shown to induce skipping of exon 6 (9). These mutations frequently occur as compound heterozygous genotypes, implying that the pathogenic effect likely relies on the combined loss of multiple functional alleles (34). While exonic mutations in CD36 are well understood (38, 39), the mechanisms of intronic mutations in affecting splicing regulatory elements remain poorly characterized. Splice-site mutations located at exon–intron boundary sequences could induce exon skipping (40). It has been previously reported that c.430-1G>C intronic mutation disrupts the highly conserved AG dinucleotide at the splice acceptor site preceding exon 6, which encodes a key extracellular domain of CD36 (20). Mutations in exon 5 commonly cause frameshifts and premature translation termination, which represent the canonical molecular characteristics of type I CD36 deficiency (15). In our study, RNA-seq quantification demonstrated marked increases in these abnormal transcripts in affected individuals. The intronic mutation-mediated exon 5/6 skipping generates an in-frame aberrant transcript in our cases; these abnormal splicing isoforms still result in the complete loss of CD36 surface expression. Although the 17-bp insertion is predicted to encode a dominant-negative truncated polypeptide, nonsense-mediated mRNA decay efficiently clears the mutant transcript, preventing functional interference with wild-type CD36. We mechanistically link the c.430-1G>C variant to aberrant splicing and loss of protein expression by FCM, thereby expanding the genetic spectrum of CD36 deficiency beyond exonic lesions. An important caveat of our work is that we failed to further dissect the biological function of the aberrant transcript arising from exon 5 skipping driven by the splice-site variant c.429 + 3*ins*G. Restricted cellular resources prevented us from carrying out supplementary *in vitro* functional tests to define the pathogenic impact of this truncated isoform, which warrants further investigation in future work.

In this study, patient 3 carried only the intronic mutations c.430-1G>C and c.429 + 3*ins*G, with no exonic mutations. RNA-seq further demonstrated that they were associated with exon 6 and exon 5 skipping, respectively. Our observations together demonstrate that intronic mutations of CD36, specifically the splicing abnormalities induced by these two mutations, severely disrupt CD36 protein structure and membrane expression, thereby constituting a key pathogenic mechanism responsible for type I CD36 deficiency. Consistent with current evidence, this finding further indicates that intronic single nucleotide mutations impair splice site recognition and induce aberrant splicing (40). Our findings further underscore the importance of including intronic regions, particularly canonical splice sites, in comprehensive molecular diagnostics for patients with CD36-related alloimmune disorders. Despite the low prevalence of only intronic-mutation-related pathogenic cases identified in our population cohort, recognition of these rare variants is crucial for accurate disease diagnosis and in-depth mechanistic interpretation of disease pathogenesis.

## Data Availability

The original contributions presented in the study are publicly available. This data can be found here: NCBI SRA, accession number PRJNA1528818.
