# Supplementary material for: Molecular basis of anti-CD36 alloimmunization: transcriptomic evidence linking intronic splice-site mutations to CD36 loss-of-function
Source: Front Immunol. 2026 Sep 28;17:1945011. doi: 10.3389/fimmu.2026.1945011 (PMC13654743; doi:10.3389/fimmu.2026.1945011)
Supplement: Supplementary file 1 [file SupplementaryFile1.docx]

**Supplementary materials**

**Supplementary Table 1 Information for every exon-exon link**

|  | conjunction | Norm01 | Norm02 | Norm03 | Norm04 | Norm05 | Norm06 | Norm07 | Norm08 | Norm09 | Norm10 | Patient 3 |  |
| --- | --- | --- | --- | --- | --- | --- | --- | --- | --- | --- | --- | --- | --- |
| 1 | exon 4-5 | 282 | 219 | 56 | 280 | 157 | 416 | 132 | 200 | 120 | 420 | 166 | normal |
| 2 | exon 4-(exon5 del2bp)-exon5 | 0 | 2 | 0 | 0 | 0 | 0 | 0 | 0 | 0 | 0 | 1 | normal-rare |
| 3 | exon 4-6 | 0 | 0 | 0 | 0 | 0 | 0 | 0 | 0 | 0 | 0 | 53 | exon5 skipping |
| 4 | exon 4-insert17bp | 0 | 0 | 0 | 0 | 0 | 0 | 0 | 0 | 0 | 0 | 5 | exon5 skipping |
| 5 | exon 4-(exon6 del89bp)-exon6 | 0 | 0 | 0 | 0 | 0 | 0 | 0 | 0 | 0 | 0 | 34 | exon5 skipping-exon6-partial skipping |
| 6 | exon 4-(exon6 del85bp)-exon6 | 0 | 0 | 0 | 0 | 0 | 0 | 0 | 0 | 0 | 0 | 10 | exon5 skipping-exon6-partial skipping |
| 7 | exon 5-6 | 338 | 276 | 82 | 361 | 303 | 475 | 175 | 302 | 133 | 511 | 0 | normal-major |
| 8 | exon5-insert17bp (with G) | 32 | 22 | 6 | 20 | 18 | 26 | 8 | 21 | 12 | 32 | 0 | normal-minor |
| 9 | exon5-insert17bp (with C) | 0 | 0 | 0 | 0 | 0 | 0 | 0 | 0 | 0 | 0 | 67 | normal-minor |
| 10 | exon 5-7 | 0 | 0 | 0 | 2 | 1 | 0 | 0 | 0 | 0 | 1 | 69 | exon6 skipping |
| 11 | exon 6-7 | 322 | 277 | 85 | 300 | 310 | 479 | 181 | 347 | 147 | 500 | 159 | normal |

**Supplementary Figure 1 Haplotype of** **patient 3 in exon 4/5/6 of CD36**

**Supplementary Figure 2** **Analysis of CD36 expression on human platelets and monocytes from patient 3 and patient 7**

The whole blood was collected from two patients. The platelets were incubated with APC-labeled CD41 and FITC-labeled CD36 and the monocytes were incubation with APC-labeled CD14 and FITC-labeled CD36, after incubation for 30 min and then analysis by flow cytometry. The CD36-positive donor as the positive control.
